# Human T-Lymphotropic Virus Type I (HTLV-1) Infection among Iranian Blood Donors: First Case-Control Study on the Risk Factors

**DOI:** 10.3390/v7112904

**Published:** 2015-11-04

**Authors:** Mohammad Reza Hedayati-Moghaddam, Farahnaz Tehranian, Maryam Bayati

**Affiliations:** 1 Research Center for HIV/AIDS, HTLV and Viral Hepatitis, Iranian Academic Center for Education, Culture and Research (ACECR), Mashhad Branch, University Campus, Azadi Sq., Mashhad 91775-1376, Iran; 2 Blood Transfusion Research Center, High Institute for Research and Education in Transfusion Medicine, Tehran 14665-1157, Iran; farahnaz_tehranian@yahoo.com (F.T.); bayati.mar@gmail.com (M.B.); 3 Razavi Khorasan Blood Transfusion Center, Mashhad 91379-13119, Iran

**Keywords:** HTLV-1 infection, risk factors, blood donors, Mashhad, Iran

## Abstract

Human T-cell lymphotropic virus type 1 (HTLV-1) infection is an endemic condition in Northeast Iran and, as such, identification of risk factors associated with the infection in this region seems to be a necessity. All the possible risk factors for HTLV-1 seropositivity among first-time blood donors were evaluated in Mashhad, Iran, during the period of 2011–2012. Blood donation volunteers were interviewed for demographic data, medical history, and behavioral characteristics and the frequencies of risk factors were compared between HTLV-1 positive (case) and HTLV-1 negative (control) donors. The data was analyzed using Chi square and *t*-tests. Logistic regression analysis was performed to identify independent risk factors for the infection. Assessments were carried out on 246 cases aged 17–60 and 776 controls aged 17–59, who were matched based on their ages, gender, and date and center of donation. Logistic analysis showed low income (OR = 1.53, *p* = 0.035), low educational level (OR = 1.64, *p* = 0.049), being born in the cities of either Mashhad (OR = 2.47, *p* = 0.001) or Neyshabour (OR = 4.30, *p* < 0001), and a history of blood transfusion (OR = 3.17, *p* = 0.007) or non-IV drug abuse (OR = 3.77, *p* < 0.0001) were significant predictors for infection with HTLV-1. Lack of variability or small sample size could be reasons of failure to detect some well-known risk factors for HTLV-1 infection, such as prolonged breastfeeding and sexual promiscuity. Pre-donation screening of possible risk factors for transfusion-transmissible infections should also be considered as an important issue, however, a revision of the screening criteria such as a history of transfusion for more than one year prior to donation is strongly recommended.

## 1. Introduction

Human T-cell lymphotropic virus type 1 (HTLV-1) was the first human retrovirus to have been discovered in 1980 [1]. Approximately 5 to 10 million people are infected with HTLV-1 worldwide [2]. HTLV-1 infection is observed throughout all parts of the world; however, Southwestern Japan, Caribbean Basin, South America, and Central Africa have been identified as being endemic regions for the virus [2]. In addition to these regions, the virus is known to be endemic in Northeast Iran especially in the cities of Mashhad and Neyshabour [3,4]. HTLV-1 is the etiological agent for adult T-cell leukemia (ATL) and HTLV-1-associated myelopathy/tropical spastic paraparesis (HAM/TSP) [2,3]. Despite this, more than 95% of infected individuals remain as asymptomatic carriers for the duration of their lives [5].

The infection can be transmitted through the transfusion of contaminated blood or blood products, unprotected sexual contact, sharing of contaminated syringes and other instruments, or via transmission from mother to child [2,6]. Risk of sero-conversion followed by transfusion is estimated to be between 40%–60% [6]. Receiving red blood cells, platelet, and whole blood compared to plasma products is also thought to be associated with a higher risk of transmission [7]; however, this risk decreases after freezing the blood for more than two weeks [6,7]. Routine screenings of blood volunteers for HTLV antibodies, along with the exclusion of high risk individuals, have contributed in reducing virus transmission through blood transfusion in endemic areas [7]. In this context, several preventive measures have been implemented by the Iranian Blood Transfusion Organization as a means to guarantee blood safety in the country and these include recording medical histories and physical examinations of the volunteers by trained physicians, encouraging people to donate blood on a regular basis, exclusion of remunerated or family replacement donations, exclusion of individuals with histories of possible risk factors such as bloodletting, tattooing, high risk sexual contact, drug abuse, *etc.* and screening for blood-borne agents including HBV, HCV, HIV, HTLV, and *Treponema pallidum* with sensitive and accurate assays [8]. The first study of Iranian blood donors in 1994 showed the prevalence of HTLV-1 infection to be 0.29%; 1.97% among Mashhadi blood donors, and 0%–0.5% in other cities [9]. After that, all donated bloods are screened for HTLV-1 and HTLV-2 antibodies in some provinces from Northeastern Iran [8]. However, the prevalence of HTLV-1 infection is still considerable among the general population (2.12%) and blood donors (0.45%) of Mashhad [3,10]. Moreover, remarkable prevalence of the infection has been reported among both blood donors and frequently blood recipients from other regions of the country as well [11,12,13,14].

To our knowledge, no survey has been performed to determine the risk factors for HTLV-1 infection in Iran. Therefore, in the current study the frequency of associated risk factors for HTLV-1 infection was investigated among blood donors in Mashhad, Northeastern Iran. Identification of individuals with these factors and excluding them from blood donation would result in a reduction of transfusion-transmitted cases of the infection.

## 2. Materials and Methods

This case-control epidemiological study was conducted among first-time blood volunteers who had been referred to blood transfusion centers of Mashhad, Iran between September 2011 and August 2013. A total of 54,436 individuals donated blood, of which 321 individuals (0.59%; 95% CI: 0.53%–0.66%) had HTLV-1 infection based on screening and subsequent confirmatory test results. The cases included 316 blood donation volunteers from Mashhad city with confirmed HTLV-1 seropositivity and the controls were selected randomly from Mashhadi donors who had shown no reactivity for HTLV-1 antibodies in screening tests. Four controls were individually matched to each case on the basis of their ages (±2 years), gender, and date and center of donation. ELISA method (EIAgen HTLV I-II Ab Kit, Adaltis S.r.l., Rome, Italy) was used as a primary detection tool of HTLV-1 antibodies and the positive results were confirmed via a Western blot analysis (MP-Diagnostics HTLV-Blot 2.4, MP Biomedicals Asia Pacific Pte. Ltd., Singapore, Singapore). All blood donors were routinely visited by the physician of the blood transfusion center before donation and checked for the presence of any blood-borne infections such as HBV, HCV, HIV, HTLV, and *Treponema pallidum*. Blood samples from the control group showed no reactivity to the abovementioned transfusion-transmitted agents.

Participants were interviewed by trained research assistants using a questionnaire on demographic and socio-economic characteristics, such as age, gender, marital status, education, income, birth place, family size, weight and height, duration of breastfeeding, medical histories including sexually-transmitted infections (STIs), blood transfusion, hospitalization, surgery, invasive diagnostic tests (endoscopy, *etc.*), dentistry procedures with bleeding (tooth extraction, gum surgery, root canal treatment, fixed dental prosthesis), suturing, acupuncture, needlestick injuries (in health settings or beauty salons), history of risky behaviors such as tattooing, cupping, tatbir (*Qama Zani*, the act of striking the head with a sword or knife until blood gushes out as a ritual), body piercing, unsafe sexual contact, drug abuse, and imprisonment.

This study was approved by The Research and Technology Deputy of Iranian Academic Center for Education, Culture and Research (ACECR) (Number: 2033-10) with regard to ethical issues. All participants in the study did so voluntarily, and signed a written consent.

All statistical analyses were performed by SPSS version 16 (Chicago, IL, USA) using a Chi square test and *t*-test. Logistic regression analysis was also performed to identify independent risk factors for HTLV-1 infection. Statistical significance was assumed for *p* values of less than 0.05.

## 3. Results

### 3.1. Demographic Characteristics

From 316 cases, 246 (77.8%) individuals aged 17–60 years had been referred for consulting and were subsequently interviewed. Moreover, 1241 controls were invited to participate in the study, of which 776 (62.5%) persons aged 17–59 years, agreed to be interviewed. As Table 1 shows, age distributions of both cases and controls were not significantly different between studied individuals and non-participants (*p* = 0.990 and *p* = 0.478, respectively). Among the case group, male to female ratio in participants was considerably less than that in non-participants (*p* = 0.004), albeit, no differences in the ratio was observed between referred and non-referred individuals from the control group (*p* = 0.329).

**Table 1:** Age and sex distributions of studied samples and non-participants from both cases and controls.

Variable	Cases			Controls		
	Studied Samples	Non-Participants	*p* Value †	Studied Samples	Non-Participants	*p* Value †
	(*n* = 246)	(*n* = 70)		(*n* = 776)	(*n* = 465)	
	No (%)	No (%)		No (%)	No (%)	
**Age (years)**			0.990			0.478
<30	52 (21.1)	15 (21.4)		193 (24.9)	132 (28.4)	
30–39	68 (27.6)	18 (25.7)		203 (26.2)	124 (26.7)	
40–49	77 (31.3)	24 (34.3)		237 (30.5)	133 (28.6)	
≥50	49 (19.9)	13 (18.6)		143 (18.4)	76 (16.3)	
**Gender**			0.004			0.329
Male	181 (73.6)	64 (91.4)		613 (79.0)	378 (81.3)	
Female	65 (26.4)	6 (8.6)		163 (21.0)	87 (18.7)	

**†** Chi square test.

**Table 2:** Demographic and socio-economic features associated with HTLV-1 infection in blood donors.

Variable	HTLV-1-Positive (*n* = 246) No (%)	HTLV-1-Negative (*n* = 776) No (%)	*p* Value †
**Age (years)**	39.1 ± 10.6 ^*^	38.3 ± 10.6 ^*^	0.25
**Gender**			
Male	181 (73.6)	613 (79)	0.08
Female	65 (26.4)	163 (21)	
**Marital status**			
Single	24 (9.8)	98 (12.6)	0.09
Married	213 (86.9)	667 (86)	
Divorced	8 (3.3)	11 (1.4)	
**Remarriage**			
Yes	19 (8.7)	25 (3.7)	0.003
No	200 (91.3)	652 (96.3)	
**Education**			
Illiterate	4 (1.6)	15 (1.9)	<0.0001
Primary school (1–5 years)	67 (27.2)	105 (13.6)	
Secondary and high school (6–12 years)	113 (45.9)	406 (52.6)	
Academic education	62 (25.2)	250 (32.3)	
**Income per month (Million Rials)**			
<5	100 (40.8)	213 (27.6)	0.001
5–9.9	113 (46.1)	401 (51.9)	
10–19.9	27 (11)	131 (17)	
≥20	5 (2)	27 (3.5)	
**Birth place**			
Mashhad (Khorasan Razavi province)	163 (66.3)	473 (61)	<0.0001
Nyshabour (Khorasan Razavi province)	20 (8.1)	28 (3.6)	
Other cities of Khorasan Razavi province	43 (17.5)	144 (18.6)	
Other provinces	20 (8.1)	131 (16.9)	
**Family size**	3.7 (1.2) ^*^	3.8 (1.3) ^*^	0.20
**BMI**			
<25	79 (33.2)	220 (39.5)	0.73
25–30	108 (45.4)	339 (47)	
≥30	51 (21.4)	163 (22.6)	
**Duration of breastfeeding**			
<6 months	8 (5.2)	47 (7.9)	0.26
≥6 moths	144 (94.7)	547 (92.1)	

BMI: Body mass index; ^*^ mean ± SD; **†** *t*-test for age and family size, Chi square test for other variables.

Demographic and socio-economic characteristics of donors are shown in Table 2. No significant difference was observed in the mean age and gender between case and control groups (*p* = 0.25 and *p* = 0.08, respectively). Cases had higher frequency of remarriage compared to controls (*p* = 0.003). The proportion of individuals with primary education or less in the case group (28.8%) was higher than those observed in the control group (15.5%; *p* < 0.0001). Furthermore, patients with the infection had significantly lower incomes compared to the controls (*p* = 0.001). According to Table 2, frequency of subjects who were born in the cities of Mashhad and Neyshabour in the case group was more than those in controls (*p* < 0.0001). In addition, no significant difference was found in the marital status (*p* = 0.09), body mass index (*p* = 0.73), family size (*p* = 0.21), and duration of breastfeeding (*p* = 0.26) between the both groups.

### 3.2. Medical Conditions

Medical histories of blood donors which were assumed to be associated with the risk of HTLV-1 infection are shown in Table 3. A history of blood or blood products transfusion in the case group was higher than that in controls (*p* = 0.005). Fifteen donors, including six cases (3.7%) and nine controls (1.5%), reported blood reception in the province of Khorasan Razavi (nearly all in Mashhad city) after the routine screening for HTLV-1 infection was started in this region (*p* = 0.07).

In addition, history of STIs was very low in both case and control groups (2.4% and 1.8%, respectively; *p* = 0.53). On the other hand, no significant differences were found in the history of hospitalization, surgery, acupuncture, suturing, invasive dental treatment, invasive diagnostic procedures, and needlestick between both groups (Table 3). Three cases of HTLV-1-HBV co-infection and one case of HTLV-1-HCV co-infection were detected but no association with HIV and *Treponema pallidum* was observed in the case group.

**Table 3:** Medical histories of blood donors associated with HTLV-1 infection.

Variable	HTLV-1-Positive	HTLV-1-Negative	*p* Value †
	(*n* = 246)	(*n* = 776)	
	No (%)	No (%)	
**Hospitalization**			
Yes	128 (51.2)	461 (57.4)	0.10
No	122 (48.8)	342 (42.6)	
**Surgery**			
Yes	98 (40.8)	327 (42.5)	0.88
No	142 (59.2)	444 (57.6)	
**Blood or blood products transfusion**			
Yes	13 (5.3)	15 (1.9)	0.005
No	233 (94.7)	761 (98.1)	
**Invasive diagnostic procedure**			
Yes	18 (7.3)	57 (7.3)	0.99
No	227 (92.7)	719 (92.7)	
**Invasive dental treatment**			
Yes	188 (76.4)	550 (71.2)	0.11
No	58 (23.6)	222 (28.8)	
**Acupuncture**			
Yes	2 (0.8)	17 (2.2)	0.16
No	244 (99.2)	759 (98.7)	
**Suturing**			
Yes	82 (33.6)	245 (31.8)	0.54
No	162 (66.4)	527 (68.3)	
**Needlestick**			
Yes	13 (5.3)	37 (4.8)	0.74
No	233 (94.7)	739 (95.2)	
**STIs**			
Yes	6 (2.4)	14 (1.8)	0.53
No	240 (97.6)	761 (98.2)	

**†** Chi square test; STIs: sexually-transmitted infections.

### 3.3. Risky Behaviors

Possible risky behaviors related to HTLV-1 infection are presented in Table 4. Frequency of cupping, piercing, tattooing, and imprisonment history did not differ between the groups; with only two cases (0.8%) and three controls (0.4%) having a history of *tatbir*. On the other hand, a history of drug abuse in the case group was significantly higher than those found in the controls (*p* < 0.0001). However, none of the subjects had stated a history of injecting drug use (IDU). Moreover, history of pre- and extra-marital sexual contact and number of lifetime sexual partners in both groups were similar (*p* = 0.17 and *p* = 0.28). On the other hand, among 22 cases with a history of pre- or extra-marital sex, 4.5% had one partner, 31.8% had two partners, and 63.6% had at least three partners. In controls with a same history, 43.6%, 27.3%, and 29.1% of 55 donors had one, two, and three or more partners, respectively (*p* = 0.002).

**Table 4:** Risky behaviors associated with HTLV-1 infection among blood donors.

Variable	HTLV-1-Positive (*n* = 246) No (%)	HTLV-1-Negative (*n* = 776) No (%)	*p* Value †
**Cupping**			
Yes	85 (34.7)	275 (35.4)	0.89
No	160 (65.3)	501 (64.6)	
**Tattooing**			
Yes	16 (6.5)	35 (4.5)	0.21
No	230 (93.5)	741 (95.5)	
**Piercing**			
Yes	47 (19.2)	155 (20.1)	0.70
No	198 (80.8)	616 (79.9)	
**Drug abuse**			
Yes	23 (9.3)	15 (1.9)	<0.0001
No	223 (90.7)	761 (98.1)	
**Pre- and extra-marital sex**			
Yes	25 (10.2)	58 (7.5)	0.17
No	219 (89.8)	715 (92.5)	
**Number of life time sexual partners**			
0	22 (9.2)	86 (11.2)	0.28
1	196 (81.7)	634 (82.3)	
≥2	22 (9.2)	50 (6.5)	
**Imprisonment**			
Yes	9 (3.7)	25 (3.2)	0.73
No	237 (96.3)	751 (96.8)	

**†** Chi square test.

### 3.4. Regression Analysis of Risk Factors Related to HTLV-1 Infection

All variables with a significant relation to HTLV-1 infection in univariate analysis were entered into the logistic regression model. As Table 5 shows, significant associations were found between the infection and low educational levels (OR = 1.64, 95% CI: 1.04–2.69), low income (OR = 1.53, 95% CI: 1.03–2.26), birth place (OR = 4.30, 95% CI: 1.91–9.67 for those born in the city of Neyshabour and OR = 2.47, 95% CI: 1.42–4.33 for Mashhad city), a history of blood transfusion (OR = 3.17, 95% CI: 1.37–7.33), and drug abuse (OR = 3.77, 95% CI: 1.79–7.55).

**Table 5:** Results from logistic regression analysis for HTLV-1 associated risk factors in blood donors.

Variable	OR	95% CI for OR	*p*-Value
**Remarriage**			
Yes	1.50	0.75–2.99	0.252
No	1.0		
**Education level**			
Illiterate or primary school (0–5 years)	1.64	1.04–2.69	0.049
Secondary and high school (6–12 years)	0.73	0.43–1.27	0.266
Academic education	1.0		
**Monthly income (Million Rials) †**			
<5	1.53	1.03–2.26	0.035
5–9.9	1.53	0.49–4.73	0.461
10–19.9	1.13	0.35–3.71	0.838
≥20	1.0		
**Birth City**			
Mashhad (Khorasan Razavi province)	2.47	1.42–4.23	0.001
Neyshabour (Khorasan Razavi province)	4.30	1.91–9.67	<0.0001
Other cities of Khorasan Razavi province	1.82	0.96–3.45	0.067
Cities of other provinces	1.0		
**History of blood transfusion**			
Yes	3.17	1.37–7.33	0.007
No	1.0		
**History of drug abuse**			
Yes	3.77	1.79–7.55	<0.0001
No	1.0		

OR: Odd ratio; CI: Confidence Interval. **†** One million Rials was approximately equal to 30 USD in the time of study.

## 4. Discussion

This study was conducted to identify possible risk factors related to HTLV-1 infection among first-time donors who referred to blood centers in Mashhad, Iran. Findings showed that the main risk factors associated with HTLV-1 infection were low educational levels, low income, being born in the cities of Mashhad and Neyshabour, and histories of blood transfusion and drug abuse.

In a study conducted on Australian blood donors during 2000–2006, the prevalence of HTLV-1 seropositivity was three per 100,000 donors, of which 5% had at least one unidentified risk factor at the time of blood donation. In general, no dominant risk factors emerged, but the major identified factors related to the infection were as follows: the country of birth and parental ethnicity (24%), sex with individual from overseas (23%), at-risk household contacts (19%), tattooing or piercing (8%), surgery or endoscopy (8%), blood product transfusion (8%), and other blood contacts, such as needle sticks (4%) [15].

In the current study, being born in the cities of Mashhad or Neyshabour showed a strong association with infection of HTLV-1. Very high prevalence of the infection in the general population of these cities had been previously reported [3,4]. However, studies conducted in other cities of Iran, had shown that HTLV-1 prevalence was not considerable [13,16].

In the present study, the case group had a significant lower education and income levels compared to the controls. It seems that low education and income levels among these people would have reduced their access to health information and may increase the prevalence of risk factors related to HTLV-1 infection, such as risky sexual behaviors or contact with contaminated blood through tattooing or piercing among these individuals. In another study on the general population of Mashhad, HTLV-1 infection was not associated with income, but the highest prevalence of the infection was observed among illiterates. Nevertheless, regression analysis showed no significant association between educational level and the infection [3]. Similarly, Custer *et al.* and Dourado *et al.* could not find any significant association between literacy and income with the infection in blood donors [17,18]. Conversely, in a case-control study by Rouet *et al.*, low educational level was identified to be a main risk factor for HTLV-1 infection among blood donors in French West Indies [19]. Furthermore, Blas *et al.* demonstrated that low literacy was indeed an important risk factor for HTLV-1 infection [20].

Breastfeeding, especially longer than six months, is one of the HTLV-1 transmission routes [21]. The current study, showed high frequencies of breastfeeding for ≥ 6 months in both groups. However prolonged breastfeeding was slightly higher in the case group compared to the controls, but this difference was not statistically significant. Lack of variability about prolonged breastfeeding might be a reason that we could not find a significant association between mode of feeding in infancy period and prevalence of the infection. Similarly, Blas *et al*. could not find a significant association between the infection and breastfeeding due to very high prevalence of breastfeeding in both HTLV-1 seropositive and seronegative individuals [20]. On the other hand, breastfeeding was reported as one of the important risk factors for HTLV-1infection among blood donors from Taiwan with an odds ratio of 4.4 [22].

As expected, this study showed that HTLV-1 infection is significantly associated with a history of blood reception. Nevertheless, there was no significant difference between the study groups if the history was limited to reception in Khorasan Razavi blood centers after 1994 when routine screening for HTLV-1 infection began in this region. A history of blood transfusion during the last year is one of the deferral criteria for blood donations in Iran and all of the blood donors had not received any blood for at least one year before the blood donation date. In a survey in Neyshabour, the proportion of the infection among individuals with a history of the transfusion was four times higher than others [4]. Additionally, in several studies on blood donors from Taiwan, Canada and Brazil a history of blood transfusion increased the risk of infection by a factor of nine to ten times [22,23,24]. On the other hand, HTLV-1 infection was not significantly associated with transfusion among healthy blood donors from Nigeria [25] and USA [17].

Based on the results of the present study, the risk of infection was not increased in individuals with a history of surgery, hospitalization, needlestick, cupping, or tattooing. Similarly, in the study conducted on the general population of Mashhad, HTLV-1 infection was not more prevalent among individuals with a history of these variables on the regression analysis [3]. In another survey among blood donors from Taiwan, a history of surgery was also not associated with the infection [22].

This study unexpectedly identified non-IDU as a risk factor for HTLV-1 infection. The frequency of non-IDU in the case group was significantly higher than in controls (9% and 2%, respectively). Likewise, Soares *et al.* showed that HTLV-1 seropositive cases in Brazilian blood donors more often used non-intravenous illegal drugs (OR = 3.3), while no significant difference was observed between HTLV-1 seropositive and seronegative groups for the frequency of intravenous drugs use [24]. In Iran, people who use opium orally or by inhalation (except heroin and crack) could donate their blood but are permanently excluded from donation if they report at least one IDU or use sharp tools for substance inhalation. It seems that the other risk factors which are not declared by the participants with a history of non-IDU or not evaluated by the researchers might have been involved in the infection of HTLV and other blood-borne viruses [26].

A history of STIs during one last year is a deferral factor for blood donation in Iran, and in the current study a history of STIs among blood donors was expectedly very low as a result. A history of extramarital sexual contact was similar among cases and controls, however, among this subgroup, cases had more lifetime sexual partner than controls. In a study conducted on injecting drug users in Mashhad prison, no association was found between HTLV-1 infection and a history of STIs and the frequency of multiple sexual partners did not differ between the HTLV-1 positive and negative groups [27]. In contrast, several studies have reported that HTLV-1 infection is associated with a history of STIs or with having multiple sexual partners [23,28,29]. Rouet *et al.* demonstrated that a history of STIs and Chlamydia seropositivity are indeed predictive factors for HTLV-1 infection among blood donors from Guadeloupe, French West Indies [19]. In addition, Melbye *et al.* showed significant correlation between having more than three partners and HTLV-1 infection among females in Guinea-Bissau, West Africa. However, they could not find a significant correlation between a history of gonorrhea and genital ulcer with HTLV-1 infection [30].

## 5. Conclusions

In summary, a history of blood transfusion, being born in the cities of Mashhad and Neyshabour, having low education and income levels, and non-IDU were significantly associated to HTLV-1 infection in first-time donors from Mashhad, Iran. Failure to detect other well-known risk factors linked to HTLV-1 infection such as breastfeeding, sexual promiscuity, and IDU could have been due, in part, to a lack of variability or the small sample size used. Various blood safety programs, such as pre-donation screening and deferral policies for blood donors with a history of possible risk factors for transfusion-transmissible infections, might also have contributed to the reduction of blood donation volunteers with these risk factors in Iran. However, a revision of the screening criteria such as a history of transfusion for more than one year prior to donation is strongly recommended.
